# The moderating role of personality traits in the relationship between interoception and somatic symptoms in youth: a predictive processing perspective

**DOI:** 10.1186/s40359-025-03705-w

**Published:** 2025-12-01

**Authors:** Henrik Eichhorn, L. Eileen Leydecker, Sebastian Brand, Stefanie M. Jungmann

**Affiliations:** 1 Department of Clinical Psychology and Psychotherapy of Childhood and Adolescence, Johannes Gutenberg University Mainz, Wallstraße 3, Mainz, 55122 Germany; 2 Department of Clinical Psychology of Childhood and Adolescence, Heidelberg University, Heidelberg, Germany

**Keywords:** Neuroticism, Perfectionism, Interoception, Interoceptive accuracy, Somatic symptoms, Predictive processing, Adolescents

## Abstract

**Objective:**

Somatic symptoms are highly prevalent in youth, but the treatment effectiveness for somatic symptom disorder is limited and the underlying mechanisms are incompletely understood. According to a predictive coding approach, low interoceptive accuracy (i.e., accuracy in perceiving internal signals from the body) may relate to somatic symptoms. So far, a potential moderating effect of personality factors on this relationship has been unexplored. Neuroticism and perfectionism could play a major role in this moderation in that they could shape expectations to be more pessimistic. This study examines the connection of neuroticism and perfectionism with interoception as well as with somatic symptoms in adolescents.

**Method:**

172 adolescents (aged 14–21) completed psychometric questionnaires assessing interoceptive accuracy, somatic symptoms, as well as levels of neuroticism and perfectionism in a cross-sectional survey.

**Results:**

Increased neuroticism was associated with reduced interoceptive accuracy (*r* = –.25, *p* = .003), whereas the relationship between perfectionism and interoceptive accuracy did not reach significance (*r* = –.17, *p* = .053). Furthermore, neuroticism and perfectionism were each associated with more somatic symptoms (*r* = .39, *p* < .001; *r* = .23, *p* = .006). Neither neuroticism nor perfectionism moderated the relationship between interoceptive accuracy and somatic symptoms (Δ*R*² = 0.06%, *p* = .794; Δ*R*² = 0.92%, *p* = .510).

**Conclusion:**

We discuss the results in the context of a predictive processing approach for the development of somatic symptoms and the need for further research.

**Supplementary Information:**

The online version contains supplementary material available at 10.1186/s40359-025-03705-w.

## Introduction

In youth, persistent and distressing somatic symptoms show a relatively high prevalence and are associated with considerable functional impairment [1]. At the same time the treatment of somatic symptom disorder shows only small to medium effects [2, 3] and mechanisms behind the emergence of somatic symptoms are not yet fully understood [4]. According to a predictive coding approach [4, 5] and empirical findings, low cardiac and self-reported interoceptive accuracy are associated with somatic symptoms (e.g., [6, 7]), whereby the perception of body symptoms could also be significantly influenced by moderator variables such as personality traits [8, 9]. The development of stressful physical complaints according to the Interoceptive Predictive Coding Model (IPCM) [4] has hardly been investigated in young people in particular. However, previous experiences with body symptoms in earlier years of life can be formative in the context of the model and there is a clear increase in body complaints in childhood and adolescence with heightened stability over years and the development of comorbid disorders [10–13]. For this reason, it is highly relevant to investigate developmental conditions such as interoceptive processes and moderating personality traits in symptom perception according to the IPCM as early as adolescence.

### Interoception and somatic symptoms

Interoception describes the perception, interpretation, and integration of internal body signals (e.g., heart rate) [14]. Individual differences in interoceptive abilities can be manifested on different levels or sub-facets of interoception as conceptualized by Suksasilp and Garfinkel [15]. In their taxonomy, self-reports should be labelled as interoceptive beliefs and they account for the consciously accessible aspect of the process, in combination with beliefs derived from other personality sources. This highlights a close interconnection between interoceptive experiences (or lack thereof) influenced by the actual strength of the afferent bodily signals to the nervous system and beliefs formed thereafter.

According to the theory of predictive processing, the brain continuously generates predictions about incoming sensory information based on prior experiences [5]. Mismatches between predicted and actual sensory input (prediction errors) cause the brain to update its predictions or modulates attention to the sensory signal. The IPCM [4] applies this framework specifically to bodily sensations. In this model, symptom perception results from the integration of prior expectations (shaped by previous experiences with bodily sensations) and current interoceptive signals. Crucially, when bodily signals are weak or ambiguous, perception may be driven more strongly by prior expectations, potentially leading to symptom experiences that are disproportionate to actual physiological changes.

The theory therefore predicts that perception of somatic symptoms can be influenced by cognitive processes (e.g., attention), the ability for interoceptive accuracy (i.e., the ability to correctly perceive bodily processes), and trait characteristics (e.g., trait negative affect). In line with this theory, interoceptive accuracy and somatic symptoms should show a negative relationship [7, 16], but overall empirical evidence is mixed [17, 18]. Although interoception has been shown to be related to various psychological processes (e.g., emotion and motivation) and psychological symptoms/disorders (e.g., eating disorders, anxiety) [14, 19, 20], this model particularly suggests a link between interoception and physical complaints/somatic symptom disorder.

In childhood and adolescence, persistent, stressful somatic symptoms such as headaches and abdominal pain are widespread. Around 10 to 25% of children and adolescents suffer from medically (largely) unexplained somatic symptoms [1, 21], often associated with considerable developmental and psychosocial impairments [22] and high socio-economic costs [23]. In this context, the present study examines bodily symptoms defined as persistent, distressing complaints for which medical evaluation yields no sufficient pathological finding. Persistent and distressing somatic symptoms usually increase with age in youth [12, 13]. In contrast to their significance, the development/maintenance of persistent/stressful somatic symptoms and in particular the IPCM in adolescence has been little studied to date.

### Neuroticism, interoception, and somatic symptoms

Neuroticism, one of the *Big Five* personality traits, accounts for transsituational consistencies in behavior and cognitions (e.g., [24–26]). This trait includes the tendency of experiencing negative affect such as anxiety, irritability, depression, and reduced emotional stability [27].

The few studies to date on the relationship between interoceptive processes and neuroticism in adulthood show different results depending on the dimension of interoception or used questionnaires. Pearson and Pfeifer [28] found, for example, that neuroticism is positively related to interoceptive awareness/attention (Body Perception Questionnaire) [29] and negatively related to trust in one’s own body (subscale of the Multidimensional Assessment of Interoceptive Awareness) [30]. Brand et al. [6] observed a significant negative correlation between self-reported interoceptive accuracy (Interoceptive Accuracy Scale) [31] and neuroticism.

Additionally, there seems to be a positive association between neuroticism and somatic symptoms in adults [24, 32, 33]. Noyes Jr. et al. [8] and van Dijk et al. [9] report that adults with unexplained somatic symptoms had higher neuroticism scores than healthy controls.

Also, in children and adolescents, neuroticism and negative affect are positively correlated with somatic symptoms, including pain [34–36]. A study by de la Barrera et al. [37] indicates that neuroticism could play a more important role in the appearance of somatic symptoms in children than in adults. Neuroticism proved to be a better predictor of somatic symptoms in a fuzzy-set qualitative comparative analysis for children than for adults.

### Perfectionism, interoception, and somatic symptoms

Although there is no uniform definition of perfectionism, current models emphasize that people with this trait tend to have high standards [38]. Frost et al. [39] describe perfectionism as a multidimensional construct, with excessive concern about making mistakes being a key component. Fassino et al. [40] revealed that perfectionist and rigid goals might result in a poorer ability to discriminate between satiety and hunger, an aspect of interoceptive accuracy.

In adolescents, perfectionism was associated with more functional somatic symptoms, both cross-sectionally and longitudinally [41]. Randall et al. [42] found a positive relationship between youth socially prescribed perfectionism (i.e., expectations convinced others have of oneself) and youth somatization. Perfectionism has also been associated with maladaptive coping strategies such as avoidance and over-engagement [43], which may contribute to an increased risk of psychosomatic complaints [44].

Given the inconsistent findings on the relationship between interoceptive accuracy, symptom reports, and interacting variables according to the IPCM, personality traits such as neuroticism and perfectionism could play a moderating role in this relationship. On a theoretical and empirical level, relationships between negative cognitions as well as negative affect and symptom reports can be identified [4, 45]. In addition to neuroticism, perfectionism, which includes the tendency to self-criticism, concern, and over-involvement [43], and similar to negative cognitions and affect, could also influence either the perception process itself as stated by Van den Bergh et al. [4], or influence the prior expectations about bodily sensations directly. Both ways, a moderator effect of personality traits should be found, because the expected negative relationship between interoceptive accuracy and symptom reports should vary depending on prior expectations *and* personality traits as processing variables. Taken together, the IPCM and relationships between interoception and distressing physical complaints in young people have been little studied. In contrast, however, the study of young age is highly relevant, as priors are often formed in young people and distressing somatic symptoms arise that lead to significant burden in everyday life.

The IPCM complements existing frameworks for understanding somatic symptoms. The biopsychosocial model [46] emphasizes the interaction of biological, psychological, and social factors in health and illness. In this context the IPCM can be viewed as specifying one mechanism through which psychological factors (e.g., personality traits shaping expectations) interact with biological processes (interoceptive signaling) to influence symptom perception [4].

### Study purpose

Understanding how personality traits shape the relationship between interoceptive processes and somatic symptoms in adolescents could inform targeted interventions and identify youth at higher risk for developing persistent symptom patterns. In the current study we adopt a dimensional perspective to examine self-reported persistent medically unexplained somatic symptoms (duration at least 6 months; to date without sufficient organic/pathological findings) in the general population. Given evidence that somatic symptom reports are dimensionally distributed [47, 48], we adopted a dimensional rather than a categorical/clinical-group approach (i.e., one based on meeting diagnostic criteria for somatic symptom disorder). Based on the theoretical foundations and empirical findings outlined, this study aims to investigate how neuroticism and perfectionism are related to interoceptive accuracy and somatic symptoms in adolescents. We expected that neuroticism is negatively related to interoceptive accuracy (*H 1.1*) and positively to somatic symptoms (*H 1.2*) in adolescents (medium-strong effects). Neuroticism was expected to moderate the relationship between interoceptive accuracy and somatic symptoms such that the higher the level of neuroticism, the stronger the negative association between interoceptive accuracy and somatic symptoms (*H 1.3*). Additionally, we expected that perfectionism is negatively correlated with interoceptive accuracy (*H 2.1*) and positively with somatic symptoms (*H 2.2*) (small effects). In addition, the higher the level of perfectionism, the greater the negative correlation should be between interoceptive accuracy and somatic symptoms in adolescents (*H 2.3*).

## Methods

### Design and sample

Our research project was a cross-sectional online questionnaire survey for adolescents aged 14 to 21 from the general population in Germany. With a targeted power of 0.95 to detect a medium effect of 0.3 with α = .05, the planned sample size resulting from G*Power [49] was *N* = 111. Assuming not all subjects complete the survey, we aimed for *N* = 140. Participants were recruited from October to December 2023 from schools in Rhineland-Palatinate, Germany and, since we did not reach enough participants this way, from universities, social media, as well as via snowball sampling through personal networks (i.e., participants and acquaintances were encouraged to share the study information with others within the target age range). For participants’ sociodemographic data of the final sample of *N* = 172^1^ see Table 1.

**Table 1:** Sociodemographic characteristics of participants

	*n*	%
Gender		
Female	114	66.3%
Male	53	30.8%
Other	4	2.4%
I do not want to answer	1	0.6%
Education		
Grammar school	82	47.7%
Secondary school plus	4	2.3%
Integrated comprehensive school	3	1.7%
Vocational school	26	15.1%
I do not go to school	44	25.6%
Other	12	7%
I do not want to answer	1	0.6%
University	34^a^	19.8% ^a^
School grade		
8	2	1.2%
9	9	5.2%
10	18	10.5%
11	38	22.1%
12	12	7%
13	33	19.2%
Other school grade	2	1.2%
I do not go to school	56	32.6%
I do not want to answer	2	1.2%

*N* = 172. Participants were on average 17.8 years old (*SD* = 2.1), aged 14 to 21 years

^a^At least. The number of collected contact details indicates that at least 34 (19.8%) of them are university students (of psychology). They stated they either did not go to school or were doing something else. Therefore, Education makes up more than 100%

### Measures

#### Interoceptive accuracy

The German Version of the Interoceptive Accuracy Scale - Version for Children and Adolescents (IAS-C) [50] assesses beliefs about their accuracy in perceiving interoceptive signals, with 20 items (e.g., “I can always perceive accurately when I am hungry.”) rated on a 5-point Likert scale (1 = *strongly disagree*, 5 = *strongly agree*). The IAS-C was used for all participants for comprehensibility; it is conceptually equivalent to the adult IAS [51]. In children aged 8 to 13 years, the IAS-C showed good internal consistency^2^ (Cronbach’s α = .86) [50]. In our sample, the scale showed a comparable score (Cronbach’s α = .84).

#### Somatic symptoms

Somatic symptoms were assessed using the Screening for Somatoform Disorders in Children and Adolescents (SOMS-KJ) [52]. The SOMS-KJ comprises 33 items assessing the presence of various medically unexplained somatic symptoms (e.g., headache) experienced during the past six months. For the purposes of the present study, only the symptom checklist component was used. Participants indicated for each symptom whether they had experienced it (1 = *yes*, 0 = *no*). A total symptom score was calculated by summing all endorsed items, with higher scores indicating a greater number of reported somatic complaints. The SOMS-KJ (designed for 11–17 years) was used across the full sample for consistency; it is conceptually equivalent to the adult SOMS [53]. In the current sample, internal consistency was also good (Cronbach’s α = .88).

#### Neuroticism

Neuroticism was measured using the subscale from Körner et al.’s NEO-Five-Factor Inventory (30-Item-Short-Version) [54], with six items (e.g., “I often feel tense and nervous”) rated on a 5-point Likert scale (1 = *strongly disagree*, 5 = *strongly agree*). In adolescents aged 14 to 16 years, Roth [55] found a Cronbach’s α = .80 for the NEO-FFI’s neuroticism scale. In our sample, Cronbach’s α was α = .87.

#### Perfectionism

Perfectionism was assessed by the German version of the Frost Multidimensional Perfectionism Scale (FMPS-D) [56]. The subscales *Concern over Mistakes*, *Doubts about Actions*, *Parental Expectations*, *Parental Criticism*, *Personal Standards*, and *Organization* are quantified by overall 35 items (e.g., “My parents set very high standards for me.”) rated on a 5-point Likert scale (1 = *does not apply at all*, 5 = *applies quite accuratel*y). The dimensions showed good internal consistency (Cronbach’s α = .71 to .92) in samples of children and adolescents [57]. Cronbach’s α = .94 in our sample indicates excellent internal consistency of the overall scale.

### Procedure

After participants had been informed about the study’s aim, nature, and scope, and had signed the consent forms, the questions were processed in the following order: query of the sociodemographic data, interoceptive accuracy, somatic symptoms, neuroticism, and perfectionism (average completion time: *M* = 9.7 min, *SD* = 3.4). Finally, participants were informed about support services for mental health problems. Students who participated could receive course credits; no other compensation.

### Data analysis

To perform statistical analyses, IBM SPSS Statistics [58] and JASP [59] were used. A significance level of 5% (α = .05) applied to all hypothesis tests.

Missing data analysis using Little’s MCAR test revealed that data were not missing completely at random (*p* < .001). The primary pattern involved 22 participants (12.8%) who completed the IAS-C and SOMS-KJ but not the NEO-FFI Neuroticism scale or the FMPS-D, likely due to survey dropout. No systematic differences were found between these 22 participants and the rest concerning age, gender, IAS-C or SOMS-KJ. As preregistered (https://osf.io/pwj5c/), if a participant answered less than three quarters of items for a given scale, their data for that scale was excluded from analyses. This resulted in varying sample sizes across analyses (see Table 2). Missing Likert scale items were replaced with the participant’s mean for that scale.

**Table 2:** Descriptive statistics of interoceptive accuracy, somatic symptoms, neuroticism, and perfectionism

	*n*	*M*	*Md*	*SD*	*Min*	*Max*
Interoceptive Accuracy	171	76.5	77	10.8	33	100
Somatic Symptoms	172	8.6	7	5.9	0	33
Neuroticism	147	2.8	2.8	1.0	1	5
Perfectionism	138	83.1	83.3	23.8	30	138.8

Interoceptive accuracy was assessed by the German Version of the Interoceptive Accuracy Scale - Version for Children and Adolescents (IAS-C) [50], somatic symptoms by the Screening for Somatoform Disorders in Children and Adolescents (SOMS-KJ) [52], neuroticism by the subscale from the NEO-Five-Factor Inventory (30-Item-Short-Version) [54], perfectionism by the German version of the Frost Multidimensional Perfectionism Scale (FMPS-D) [56].

To test whether perfectionism and neuroticism affect the strength of the association between interoceptive accuracy and somatic symptoms, moderation analyses using Hayes’ PROCESS macro [60] were conducted. Bootstrapping with 10,000 samples was used to calculate confidence intervals along with heteroscedasticity-consistent standard errors (HC3) [61]. As a sensitivity analysis, all bivariate correlations were repeated with the sample restricted to participants aged ≤ 18 and > 18 years to address potential developmental heterogeneity between mid-adolescence and emerging adulthood.

## Results

### Descriptive statistics

Table 2 Contains descriptive statistics of the four scales. Somatic symptoms that were reported the most were headache (80.23%), fatigue/weakness (70.93%), stomachache (64.53%) and back pain (62.21%). Interoceptive accuracy and somatic symptoms were not associated significantly in our sample (*r* = –.14, *p* = .075, 95% CI [–.28, .01]), whereas the correlation between neuroticism and perfectionism was high (*r* =.60, *p* < .001, 95% CI [.48, .70]). As a supplementary analysis, Table A (see Appendix) shows a heat map of all correlation coefficients including significance between the four examined variables.

### Hypothesis testing

For *H 1.1*, a significantly negative correlation of *r* = –.25 (*p* = .003, 95% CI [–.39, − .09]) was found between neuroticism and interoceptive accuracy.

**Fig. 1:**
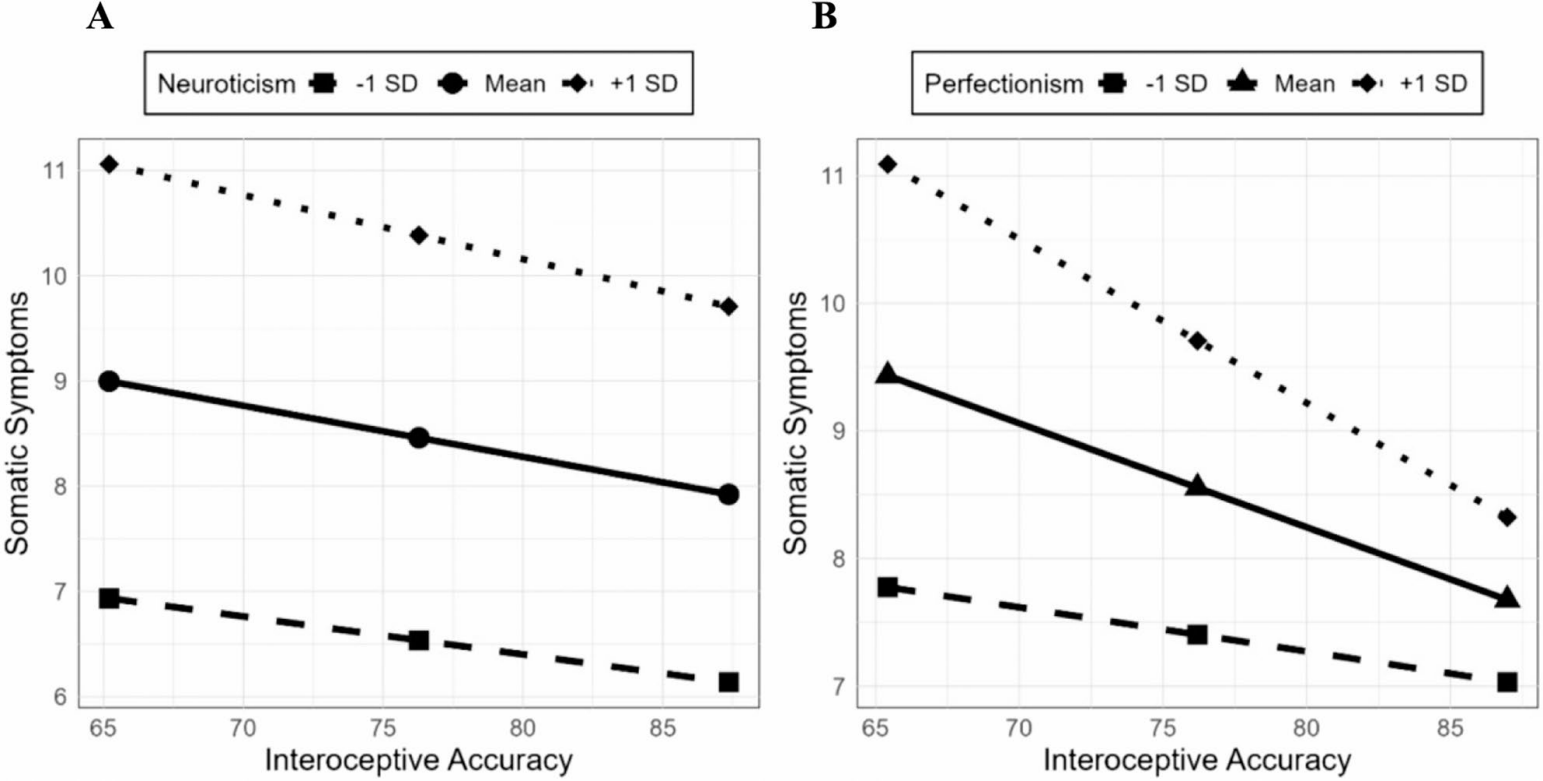
Plots showing the relation of interoceptive accuracy with somatic symptoms for − 1 *SD* (■), *M* (●, ▲) and + 1 *SD* (◆) in neuroticism (**A**) and perfectionism (**B**)

For *H 1.2*, there was a significantly positive correlation between neuroticism and somatic symptoms in adolescents, with *r* = .39 (*p* < .001, 95% CI [.25, .52]). For *H 1.3*, the overall regression model including interoceptive accuracy, neuroticism, and their interaction was significant, *F*(3, 142) = 8.51, *p* < .001, with a medium variance explanation of 15.67%, indicating these variables collectively explained variance in somatic symptoms. However, the interaction term (testing moderation) did not significantly improve model fit, Δ*R²* = 0.06%, *F*(1, 142) = 0.07, *p* = .794, 95% CI [–.11, .07] (see Fig. 1A). The model’s explanatory power was driven primarily by the main effect of neuroticism rather than the moderation.

For *H 2.1*, a non-significant correlation between perfectionism and interoceptive accuracy of *r* = –.17 (*p* = .053, 95% CI [–.32, .00]) was found.

For *H 2.2*, there was a significantly positive correlation between perfectionism and somatic symptoms, *r* = .23, *p* = .006, 95% CI [.07, .38].

For *H 2.3*, the overall regression model including interoceptive accuracy, perfectionism, and their interaction was also significant, *F*(3, 134) = 4.38, *p* = .001, with a low variance explanation of 8.29%, but the interaction term testing moderation was not significant, Δ*R²* = 0.92%, *F*(1, 134) = 0.44, *p* = .510, 95% CI [–.01, .00] (see Fig. 1B). The model’s variance explanation was attributable to main effects rather than moderation.

Sensitivity analyses restricted to participants aged ≤ 18 (*n* = 108) and > 18 years (*n* = 62) yielded substantively similar results in terms of size and direction, while not all relationships were significant likely due to smaller sample sizes. This supports the robustness of findings across the full age range.

## Discussion

Based on inconsistent findings, (mostly) from adulthood, and the IPCM [4], this study aimed to investigate the relationships between self-reported interoceptive accuracy, personality traits (neuroticism, perfectionism), and body symptoms in youth. We found significant associations between personality traits and both interoceptive accuracy and somatic symptoms, but no evidence that personality traits moderated a possible interoception-symptom relationship. To our knowledge, this is the first study to investigate the moderating influence of personality factors on the relationship between interoceptive accuracy and body symptoms in adolescents.

All but one of the hypothesized bivariate relationships showed significant correlations. There was a significant positive relation between trait neuroticism and somatic symptoms while the trait was negatively associated with self-reported interoceptive accuracy in our sample. The same pattern was found for perfectionism except for the relation with interoceptive accuracy, which missed to reach statistical significance (*p* = .053), while the two personality traits showed a considerable overlap (*r* = .60). Though interoceptive accuracy and somatic symptoms show a theory-compliant descriptive negative correlation (albeit not significant), analyses performed to test for a possible moderating effect of the personality traits on the relationship between interoceptive accuracy and somatic symptoms both delivered non-significant results, even though the sample was chosen due to considerations of the effect might be stronger in younger people. Both the IAS-C and SOMS-KJ were developed for children and adolescents but were used across our sample (14–21 years) to ensure consistency. They differ from the adult versions mainly in simplified wording and minor format adaptations, while assessing conceptually equivalent constructs. The relatively wide age range may have introduced developmental heterogeneity, potentially obscuring stage-specific mechanisms if moderation effects differ across developmental periods.

Our sample reported an average of 8.6 different somatic symptoms (*SD* = 5.9; range: 0–33), comparable to community adolescent samples also in terms of most common symptoms [62]. The observed associations between somatic symptoms and personality traits align with existing literature [24, 33, 41]. However, for interoceptive accuracy, to our knowledge no research has so far been made on its relation to personality traits in an adolescent population. Compared with existing literature on adults, our findings here show similar patterns on that relation [6, 40]. Given the absence of a moderation effect, the question needs to be discussed whether the reasons lie in the methodological assessment or whether the substantive assumption is not (fully) correct (e.g., another interoceptive dimension).

For example, it is possible that the moderation can be found with another dimension of the model of [15], i.e. via the process of interoceptive attribution, which highlights the interplay between interoception, attributive beliefs, and possible resulting pathological phenomena. It could also be assumed that other moderators play a role in the relationship between interoceptive accuracy and physical complaints (e.g., emotion regulation) or that it is more of a mediating relationship. Methodologically, while the IAS-C captures meaningful variance in interoceptive accuracy, as with any self-report measure, it integrates both actual interoceptive abilities and beliefs about those abilities [15]. This is a fundamental challenge in psychological assessment – similar to measuring any internal construct through self-report – but does not invalidate the measure’s utility in capturing individual differences in the construct of interest. Future research incorporating complementary behavioral measures (e.g., heartbeat perception tasks) alongside self-report would provide convergent evidence and allow separation of belief-based and performance-based components of interoception. Systematic missingness was observed, with 12.8% of participants completing symptom and interoception measures but not personality scales, likely reflecting survey dropout. This reduced sample size for moderation analyses and may have affected statistical power. Collectively, these methodological limitations – measurement approach, sample attrition, and design constraints – may have obscured moderation effects that could emerge under different study conditions, though theoretical alternatives (e.g., mediation, different interoceptive dimensions) also warrant consideration.

Another reason for the missing moderation effects may be that we only assessed the number of different somatic symptoms rather than their subjective severity. In the context of the IPCM, it may be more the intensity of symptom severity (e.g., intensity scale; Likert scale) that is influenced by an interaction of interoceptive accuracy and personality traits (as opposed to the number of symptoms). Furthermore, in the predictive processing framework not only the mean point values of the constructs are relevant when representing distributions. Their precision also needs to be accounted for when trying to empirically test the theory. Precision reflects how strongly expectations versus current signals influence perception, and can vary dynamically across contexts and signal strength [5]. Estimating these precision parameters requires experimental designs with repeated measures or systematic manipulation of signal ambiguity, which was beyond the scope of our cross-sectional study. Our approach captures stable individual differences in interoceptive accuracy and personality traits as relevant components of the IPCM, providing a foundation for future studies incorporating dynamic precision estimation. Longitudinal studies with multiple assessments across varying contexts would be of great interest for examining precision dynamics. We want to further underline that the predictive processing approach to somatic symptoms is promising but urgently requires further empirical testing. Besides, no statements about causality can be inferred from the findings of the present study, as only correlations and moderation analyses were calculated. A further limitation of the present study is the absence of standardized assessments of functional disability, anxiety, and depression, which may be relevant for understanding individual differences in somatic symptoms and personality traits. Socioeconomic status (including parental education, school type, and family income), as well as racial and ethnic identity, were not assessed, which restricts the consideration of possible sociodemographic influences on somatic symptom reporting. Additionally, potential sex/gender differences were not examined, but warrant investigation in future research with larger samples, particularly given the domain-specific nature of sex differences in interoceptive accuracy [63]. We note that our unequal gender distribution restricts generalizability. The use of snowball sampling through personal networks may limit the generalizability of the findings.

## Conclusions

So far, the treatment of somatic symptom disorders has been mediocre at best [2, 3]. Understanding the mechanisms behind the emergence and maintenance of somatic symptom distress will help to improve and extend current interventions. Personality traits were associated with the perception of somatic symptoms, which may be relevant for future theoretical models or intervention research. It is possible that personality traits can influence the priors in IPCM, whereby a tendency towards negative affect or high standards could explain the altered or distorted perception, especially with mild/ambiguous sensory input. This study provides the first empirical evidence that personality traits, especially neuroticism, are associated with both interoceptive accuracy and symptom reporting in young people. It is promising for research to further investigate the exact interaction of these variables.

## Supplementary Information

Supplementary Material 1.

## Data Availability

The datasets generated and analysed during the current study are not publicly available due to the authors effort to treat data stemming from minors with special care and responsibility, but are available from the corresponding author on reasonable request.

## Abbreviations

IPCM: Interoceptive Predictive Coding Model
IAS-C: Interoceptive Accuracy Scale – Version for Children and Adolescents
SOMS-KJ: Screening for Somatoform Disorders in Children and Adolescents
NEO-FFI-30: NEO-Five-Factor Inventory (30-Item-Short-Version)
FMPS-D: German version of the Frost Multidimensional Perfectionism Scale

## Footnotes

1 As described, we initially planned to recruit *N* = 140 participants. However, we exceeded our recruitment target due to unexpectedly high participant interest in the second recruitment phase, allowing us to include more participants.

2 In this paper, we use Blanz’s [48] guidelines for the interpretation of Cronbach’s α.
