# Supplementary material for: The moderating role of personality traits in the relationship between interoception and somatic symptoms in youth: a predictive processing perspective
Source: BMC Psychol. 2025 Dec 1;13:1324. doi: 10.1186/s40359-025-03705-w (PMC12670852; doi:10.1186/s40359-025-03705-w)
Supplement: Supplementary file 1 — Supplementary Material 1. [file 40359_2025_3705_MOESM1_ESM.docx]

# Appendix.

| **Table A** |  |
| --- | --- |
| *Heat Map: Product Moment Correlations Between Interoceptive Accuracy, Somatic Symptoms, Neuroticism, and Perfectionism* | |
| 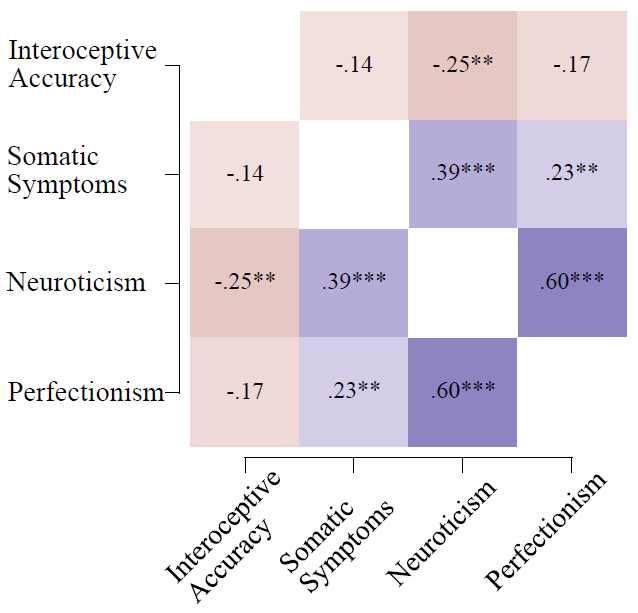 | |
| *Note*. 95% CI [–0.28, 0.01] for *r*_Somatic Symptoms, Interoceptive Accuracy_. 95% CI [–0.39, –0.09] for *r*_Neuroticism_*_,_* _Interoceptive Accuracy_. 95% CI [–0.32, 0.00] for *r*_Perfectionism_*_,_* _Interoceptive Accuracy_. 95% CI [0.25, 0.52] for *r*_Neuroticism_*_,_* _Somatic Symptoms_. 95% CI [0.07, 0.38] for *r*_Perfectionism_*_,_* _Somatic Symptoms_. 95% CI [0.48, 0.70] for *r*_Perfectionism_*_,_* _Neuroticism_.  * *p* < .05, ** *p* < .01., *** *p* < .001. | |

**Figure A**

*Histogram depicting how often each symptom count was reported on the SOMS-KJ*

**
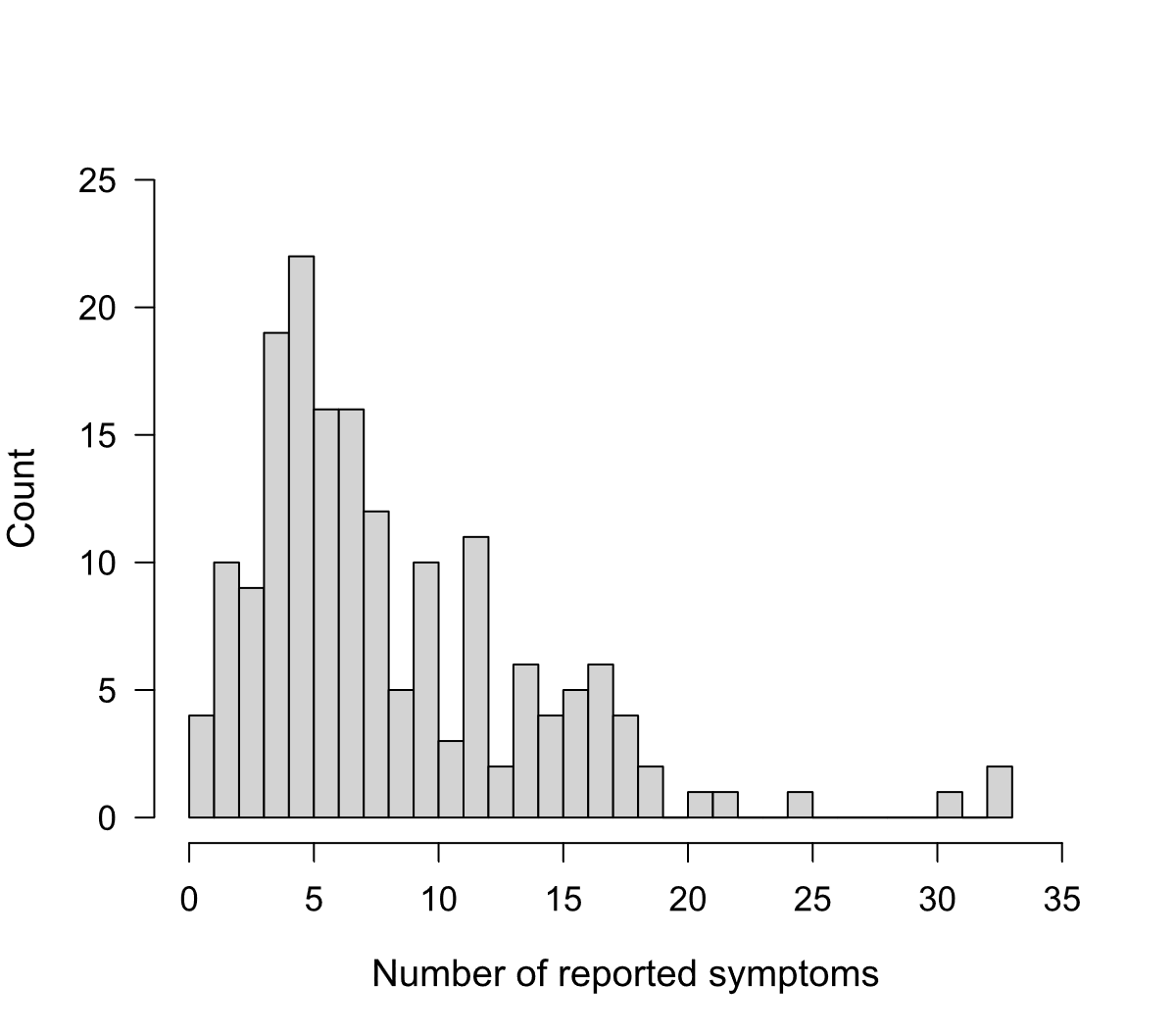
**

*Note.* Total *N* = 172.
